# Invariant Natural Killer T-cells and their subtypes may play a role in the pathogenesis of endometriosis

**DOI:** 10.1016/j.clinsp.2022.100032

**Published:** 2022-05-13

**Authors:** Frederico J.S. Correa, Marina Paula Andres, Tainá Pezzin Rocha, Ana Eduarda Z. Carvalho, Thiago P.A. Aloia, Marcus V.N. Corpa, Esper G. Kallas, Cristóvão L.P. Mangueira, Edmund C. Baracat, Karina I. Carvalho, Mauricio S. Abrão

**Affiliations:** a Endometriosis Section, Divisão de Clínica Ginecológica, Hospital das Clínicas, Faculdade de Medicina, Universidade de São Paulo (HCFMUSP), São Paulo, SP, Brazil; b Gynecology and Obstetrics Department, Faculdade de Medicina da Universidade de Brasília (UnB), Campus Universitário Darcy Ribeiro, Brasília, DF, Brazil; c Gynecologic Division, BP ‒ A Beneficência Portuguesa de São Paulo, São Paulo, SP, Brazil; d Laboratório de Pesquisa e Desenvolvimento Peter Murányi, Hospital Israelita Albert Einstein, São Paulo, SP, Brazil; e Laboratório de Medicina Diagnóstica e Preventiva, Hospital Israelita Albert Einstein, São Paulo, SP, Brazil; f Faculdade de Medicina, Universidade de São Paulo, São Paulo, SP, Brazil

**Keywords:** Deep endometriosis, Invariant natural Killer T-cells, Cytokines, Flow cytometry, Confocal microscopy, NK, Natural Killer, iNKT, invariant Natural Killer T, PBMCs, Peripheral Blood Mononuclear Cells, VAS, Visual Analogic Scale

## Abstract

**Objective:**

To evaluate the frequencies of iNKT cells and their subsets in patients with deep endometriosis.

**Methods:**

A case-control study was conducted between 2013 and 2015, with 73 patients distributed into two groups: 47 women with a histological diagnosis of endometriosis and 26 controls. Peripheral blood, endometriosis lesions, and healthy peritoneal samples were collected on the day of surgery to determine the frequencies of iNKT cells and subtypes via flow cytometry analysis.

**Results:**

The authors observed a lower number of iNKT (*p* = 0.01) and Double-Negative (DN) iNKT cells (*p* = 0.02) in the blood of patients with endometriosis than in the control group. The number of DN iNKT IL-17^+^ cells in the secretory phase was lower in the endometriosis group (*p* = 0.049). There was an increase in the secretion of IL-17 by CD4^+^ iNKT cells in the blood of patients with endometriosis and severe dysmenorrhea (*p* = 0.038), and severe acyclic pelvic pain (*p* = 0.048). Patients with severe dysmenorrhea also had a decreased number of CD4^+^ CCR7^+^ cells (*p* = 0.022).

**Conclusion:**

The decreased number of total iNKT and DN iNKT cells in patients with endometriosis suggests that iNKT cells play a role in the pathogenesis of endometriosis and can be used to develop new diagnostic and therapeutic agents.

## Highlights

• The frequency of iNKT cells in general and their subtype double-negative are related to endometriosis.
• The expression of IL-17 and CCR7 by iNKT cells are related to endometriosis-associated pain symptoms.
• iNKT cells are numerically and functionally altered in women with endometriosis.

## Introduction

Endometriosis is an inflammatory disease characterized by the presence of endometrial glands and/or stroma outside the uterus, with an estimated prevalence of 10% in women of reproductive age.^1^ Its main symptoms are dysmenorrhea, deep dyspareunia, chronic pelvic pain, and infertility. These clinical manifestations are heterogeneous and not always compatible with the severity or stage of the disease.^2^

Several studies have demonstrated the importance of the immune system in the pathogenesis of endometriosis. Disturbances in immunological homeostasis can facilitate implantation, proliferation, and angiogenesis of endometrial tissue in the peritoneum.^3^^,^^4^ Endometriosis is also associated with changes in the frequencies of lymphocyte populations, altered cytotoxicity of Natural Killer (NK) cells, and the Th1 response induced by Th2-type pro-inflammatory and anti-inflammatory cytokines.^5^, ^6^, ^7^

In recent years, the importance of invariant Natural Killer T (iNKT) cells in the control of Th1, Th2, and Th17 immune responses and their relation to certain diseases has been demonstrated.^8^, ^9^, ^10^ iNKT cells are a subclass of T-lymphocytes that express NK cell markers such as CD161 and an invariant T-Cell Receptor (TCR) α/β with a restricted repertoire. These cells constitute 0.2% of the total T-cells in the peripheral blood.^14^ Given the essential role of iNKT cells in inflammatory, infectious, and autoimmune diseases,^15^ the authors hypothesized that iNKT cells could secrete cytokines and modulate the inflammatory response in patients with endometriosis. However, only a few studies on iNKT and endometriosis have been published.^11^, ^12^, ^13^

The objective of this study was to evaluate the association between iNKT cells and their subsets with endometriosis. The secondary objectives include the evaluation of cytokine profiles and the correlation between the frequency of iNKT and pain symptoms.

## Methods

### Study design

A prospective study was conducted between 2013 and 2015 at the Endometriosis Clinic, Hospital das Clínicas, Faculdade de Medicina, Universidade de São Paulo, São Paulo, Brazil, and the study was approved by its institutional ethics committee (CAPPesq 235869/13), and it is in accordance with the Helsinki Declaration. Forty-seven women aged 18–49 years with regular menstrual cycles who underwent laparoscopic surgery for the treatment of deep endometriosis with histological confirmation were included in the endometriosis group. Twenty-six healthy women without endometriosis upon laparoscopy for tubal ligation were included in the control group. Patients who received hormonal treatment in the past three months or had autoimmune diseases were excluded. Written informed consent was obtained from all participants.

Clinical data and grading of dysmenorrhea, deep dyspareunia, acyclic pelvic pain, cyclic dyschezia, and cyclic dysuria were obtained from all included patients using a Visual Analog Scale (VAS) from 0 to 10. The authors considered severe pain as having VAS scores between 7 and 10 and mild pain as having VAS scores between 0 and 6.

During laparoscopy, before installation of the pneumoperitoneum, endometrial biopsies for menstrual cycle phase confirmation were obtained using a Novak curette, and blood samples were also collected. A complete evaluation of the pelvis and staging of endometriosis according to the American Society of Reproductive Medicine (ASRM, 1996) was performed, and all suspected lesions were completely resected. Samples of endometriosis lesions were obtained and stored in liquid nitrogen at the Research Center of Hospital Israelita Albert Einstein until subsequent analysis.

### Flow cytometry

Peripheral Blood Mononuclear Cells (PBMCs) were thawed, isolated, washed, and counted. Their viability was assessed using a Countess® automated cell counter (Invitrogen, Carlsbad, CA, USA), and they were frozen in liquid nitrogen until use.

Surface immunostaining of PBMCs was performed at 20–30°C for 30 min in 96-well V-bottom plates (Nunc®, Thermo Fisher Scientific, Waltham, MA, USA) using the following antibodies: CD3 (clone UCHT1, Beckman Coulter, Brea, CA, USA), Vβ11 (clone C21, Beckman Coulter), and Vα24 (clone C15, Beckman Coulter); CD4 (clone RPA-T4, Biolegend, San Diego, CA, USA), CD8 (clone RFT-8, Biolegend), CD14 (clone M5E2, Biolegend), CD19 (clone HIB19, Biolegend), CD25 (clone M-A251, Biolegend), and CCR7 (clone 3D12; BD Biosciences, San Jose, CA, USA). Amine Aqua dye (Invitrogen, Thermo Fisher Scientific) was used to exclude dead cells from all samples. Samples were washed and fixed with formaldehyde (Sigma-Aldrich, Merck KGaA, Saint Louis, MO, USA) before flow cytometry data acquisition.

To measure cytokine production, PBMCs were incubated in the presence of their cognate iNKT-specific agonist α-galactosylceramide (αGalCer). After incubation for 1h at 37°C under a 5% CO_2_ atmosphere_,_ monensin (Golgi Stop, BD Biosciences) or Brefeldin A (Golgi Plug, BD Biosciences) was added. After incubation for 18h, the cells were washed and incubated with monoclonal antibodies against surface antigens. After incubation, the cells were washed, fixed, and permeabilized using reagents from Life Technologies (Thermo Fisher Scientific), according to the manufacturer's instructions. Cells were incubated with antibodies against IL-6 (clone MQ2-13A5, BD Biosciences), IL-10 (clone JES3-1931, Beckman Coulter), and IL-17 (clone: BL168; Biolegend). The cells were then analyzed using an LSR Fortessa flow cytometer (BD Biosciences). All samples were acquired using FACSDiva software (BD Biosciences), and data were processed using FlowJo software (version 9.9, Tree Star).

### Immunofluorescence staining and confocal microscopy

For immunofluorescence staining, the endometriosis lesions were cut into 5 µm sections, stained, and analyzed by a pathologist to define the endometriosis lesions and healthy areas in the sections. Afterward, the sections on the slides were dewaxed and subjected to an epitope retrieval step via incubation in sodium citrate buffer for 15 min in a microwave. Next, the slides were incubated overnight at 4°C with mouse anti-human CD1d (BD Biosciences) and rabbit anti-human CD3 polyclonal (Abcam, Cambridge, UK) primary antibodies. The slides were then washed and incubated with secondary antibodies, Alexa 488 anti-mouse and Alexa 568 anti-rabbit (Life Technologies, Thermo Fisher Scientific) for 2h at 20–30°C. Next, the sections were washed and incubated with anti-CD4 antibody (clone RPA-T4) for 2h, and then incubated with DAPI for 5 min. Slides were mounted with Prolong Gold Antifade Reagent (Life Technologies, Thermo Fisher Scientific). Images were acquired using an LSM 710 confocal microscope (Carl Zeiss, Jena, Germany). The sections were imaged using ZEN 2012 SP2 software (Black, 64-bit, Release Version 11.0, Carl Zeiss).

### Statistical analysis

Sample size calculation was performed assuming 95% confidence and 80% power. Previous studies have shown that the percentage of iNKT cells in the blood of normal women ranges from 0.1% to 2%, with a standard deviation of 0.27%; therefore, five women in each group would be required to identify a mean difference of 0.5% iNKT cells between women with and without endometriosis.^16^ iNKT cell frequencies and interleukin levels in the peripheral blood were compared between groups using the Mann-Whitney test. Comparisons between groups regarding symptoms, stage of endometriosis, and menstrual cycle phase were also performed using the Mann-Whitney test. The analyses were performed using SPSS software (version 20.0, International Business Machines Corporation, Sao Paulo, SP, Brazil). Statistical significance was set at p≤0.05.

## Results

### Clinical characteristics of patients

The mean age of patients was similar between the endometriosis (34.3 ± SD 6.2 years) and control groups (34.5 ± 4.6 years; *p* = 0.87). The mean Body Mass Index (BMI) was higher in the control group compared to the endometriosis group (24.1 ± 3.1 kg/m^2^ vs. 22.5 ± 3.3 kg/m^2^; *p* = 0.046). Patients with endometriosis presented with a higher incidence of dysmenorrhea (63.8% vs. 11.5%; *p* < 0.001) and cyclic dyschezia (14.9% vs. 0%; *p* = 0.046) than those without endometriosis. There was no significant difference in acyclic pelvic pain, dyspareunia, or cyclic dysuria between the groups. No differences were observed in the menstrual phases between the groups (Table 1).

**Table 1:** Demographic and clinical characteristics in endometriosis and control group.

	Group			
	Control	Endometriosis	Total	
Characteristic	(*n* = 26)	(*n* = 47)	(*n* = 73)	*p*
Age (years)	34.5±4.6	34.3±6.2	34.4±5.7	0.876^c^
BMI (Kg/m²)	24.1±3.1	22.5±3.3	23±3.3	**0.046**^c^
Dysmenorrhea, n (%)	3 (11.5)	30 (63.8)	63.8 (33)	**<0.001**^a^
Severe acyclic pelvic pain, n (%)	2 (7.7)	9 (19.1)	19.1 (11)	0.308^b^
Severe dyspareunia, n (%)	1 (3.8)	10 (21.3)	21.3 (11)	0.084^b^
Severe cyclic dyschezia, n (%)	0 (0)	7 (14.9)	14.9 (7)	0.046^b^
Severe dysuria, n (%)	0 (0)	1 (2.1)	2.1 (1)	>0.999^b^
Menstrual phase, n (%)				0.067^a^
Proliferative	14 (53.8)	15 (31.9)	31.9 (29)	
Secretory	12 (46.2)	32 (68.1)	68.1 (44)	

Mean ± standard deviation; n, number; %, percentage; BMI, Body Mass Index.

Pain ≥ 7 in visual analog scale was considered severe.

a Chi-Square test.

b Fisher's exact test.

c Student's *t*-test.

### Frequency of iNKT cells in peripheral blood

**Fig. 1:**
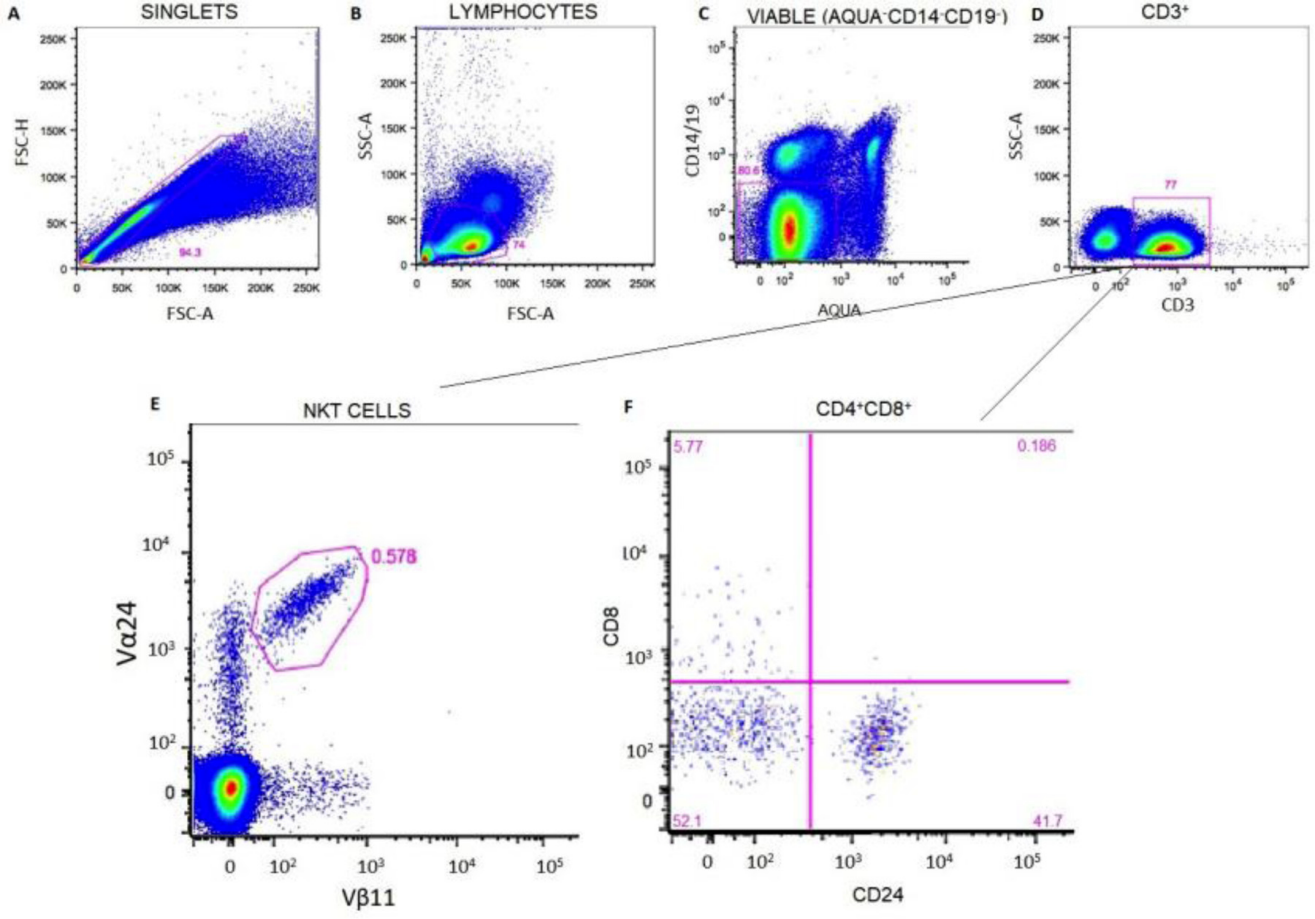
Gating strategy for the identification and analysis of iNKT cells. This figure shows the standardized gating strategy used for flow cytometry: (A) singlets, (B) mononuclear cells (excluding debris (FSClow/SSClow), (C) viable lymphocytes, (D) CD3 positive cells, (E) NKT cells (CD3-CD56^+^ cells), and (F) CD4^+^ CD8^+^ cells.

**Fig. 2:**
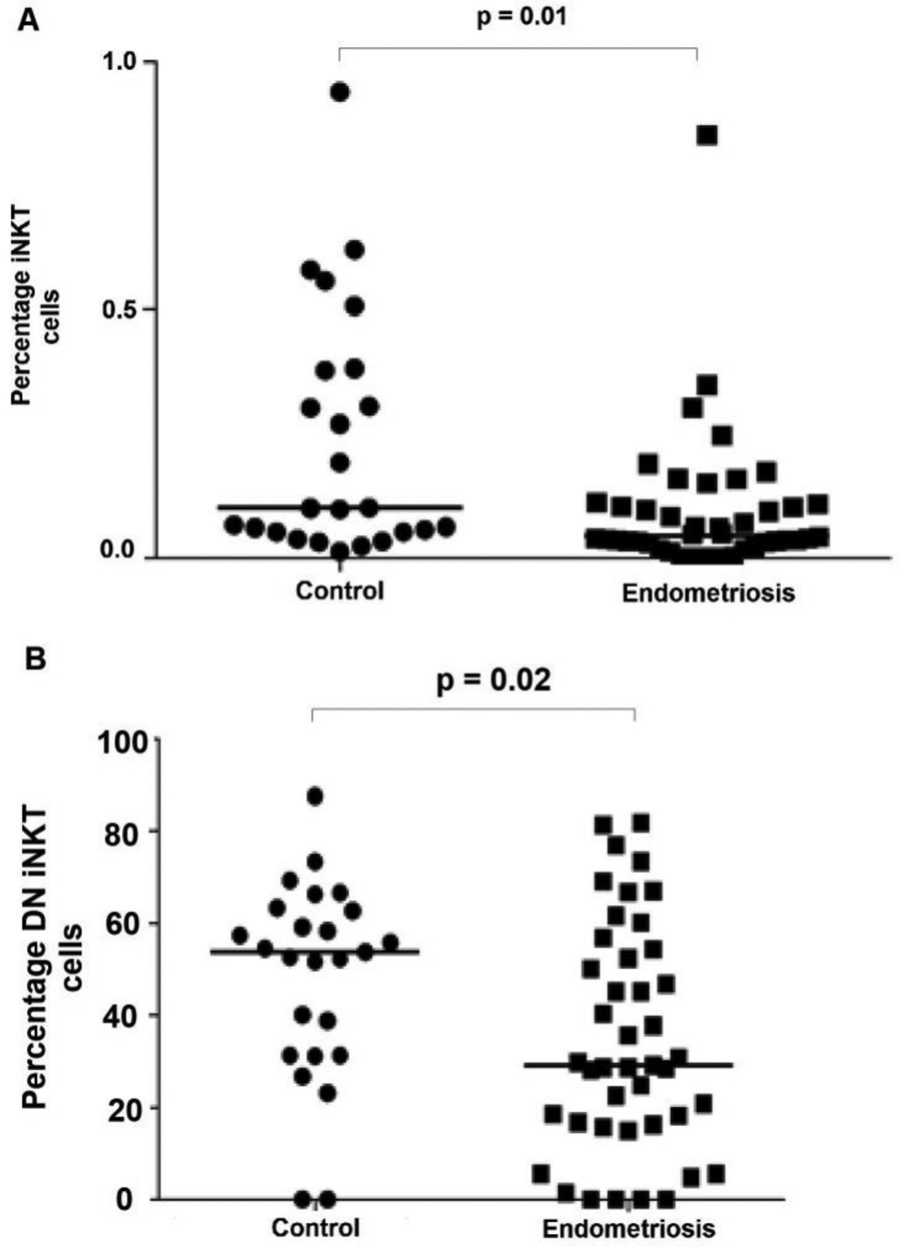
Distribution of frequencies of iNKT cells between the endometriosis and control groups. (A) Figure shows a decrease in the total number of iNKT cells in the peripheral blood of patients with endometriosis compared to those without the disease. (B) Figure shows that the number of DN iNKT cells was decreased in the peripheral blood of patients with endometriosis compared to those without the disease.

The number of iNKT cells was determined through the co-expression of surface markers Vα24 and Vβ11, as shown in the gating strategy in Fig. 1, using multiparametric flow cytometry. The authors observed a decrease in the total number of iNKT cells in the peripheral blood of patients with endometriosis compared to those without the disease (0.17 ± 0.55 vs. 0.23 ± 0.25; *p* = 0.01) (Table 2, Fig. 2A).

**Table 2:** Total number of iNKT cells and its subsets in the peripheral blood of patients with endometriosis and controls.

	Group			
	Control	Endometriosis	Total	
Variable	(*n* = 26)	(*n* = 47)	(*n* = 73)	*p*
Total iNKT (mean ± SD)	0.23±0.25	0.17±0.55	0.2±0.46	**0.010**
iNKT CD4+	32.2±21	37±27.4	35.2±25.1	0.673
iNKT CD4+/ CCR7	31.6±31	35.6±30.6	34.1±30.6	0.439
iNKT CD4+/ CD25	25.8±18.5	33.5±23.1	30.8±21.8	0.168
iNKT DN	48.2±21.4	34.6±24.5	39.6±24.2	**0.020**
iNKT DN/ CCR7	6.76±14.08	11.18±15.87	9.54±15.27	0.801
iNKT DN/ CD25	33.8±29.8	39.7±31.8	37.4±30.9	0.416

Mann-Whitney test; SD, Standard Deviation.

The authors used the gating strategy shown in Fig. 1 to identify all iNKT subsets: CD4^+^, CD8^+^, double-positive (DP) CD4^+^CD8^+^, and double-negative (DN) CD4^−^CD8^−^ cells.^17^^,^^18^ The number of DN iNKT cells was decreased in the peripheral blood of patients with endometriosis compared to those without endometriosis (34.6 ± 24.5 vs. 48.2 ± 21.4; *p* = 0.02), (Table 2, Fig. 2B). The number of other iNKT subsets did not differ between the endometriosis and control groups (Table 2).

**Fig. 3:**
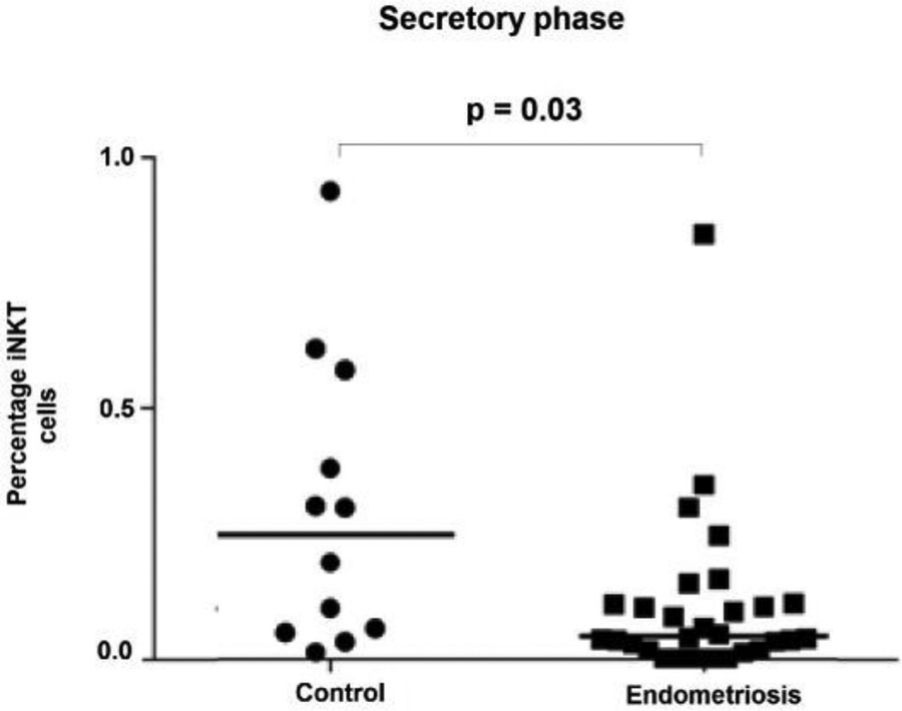
Distribution of frequencies of iNKT cells in the secretory menstrual phase between groups showing that patients with endometriosis had significantly lower numbers of total iNKT cells compared to the control group.

Analysis of the menstrual cycle phases indicated that in the secretory phase, patients in the endometriosis group had lower numbers of total iNKT cells compared to the control group (median 0.05 [range: 0–3.61] vs. 0.25 [0.01–0.94]; *p* = 0.03) (Fig. 3). There were no statistically significant differences in a total number of iNKT cells between menstrual cycle phases in the endometriosis group (Table 3).

**Table 3:** Total number of iNKT cells its subsets, and cytokine expression in the peripheral blood of patients with endometriosis and controls, according to the menstrual cycle phase.

		Group		
Variable	Menstrual cycle phase	Control	Endometriosis	*p*
iNKT	Proliferative	0.07 (0.02‒0.56)	0.04 (0‒0.19)	0.128
	Secretory	0.25 (0.01‒0.94)	0.05 (0‒3.61)	0.030
	p	*0.295*	*0.880*	
iNKT CD4+	Proliferative	31.1 (7.2‒73.1)	30.1 (0‒100)	0.734
	Secretory	28.6 (0‒69.6)	34.1 (0‒81.1)	0.747
	p	*0.936*	*0.479*	
iNKT CD4+/CCR7	Proliferative	41.3 (0‒83.1)	53.8 (0‒77.5)	0.470
	Secretory	0 (0‒92.3)	40.6 (0‒83.5)	0.460
	p	*0.316*	*0.368*	
iNKT CD4+/CD25	Proliferative	33.2 (0‒58.6)	44.6 (0‒60)	0.022
	Secretory	25.6 (0‒57.7)	30 (0‒81.7)	0.555
	p	*0.674*	*0.121*	
iNKT DN	Proliferative	55.8 (23.1‒87.5)	28.8 (0‒73.3)	0.011
	Secretory	53.1 (0‒69.2)	30.6 (0‒81.7)	0.357
	p	*0.611*	*0.542*	
iNKT DN/CCR7	Proliferative	0.5 (0‒36.6)	4.27 (0‒48.6)	>0.999
	Secretory	0.1 (0‒45.5)	6.9 (0‒60)	0.745
	p	*0.689*	*0.897*	
iNKT DN/CD25	Proliferative	46.2 (0‒76.2)	62.5 (0‒100)	0.101
	Secretory	17 (0‒72.3)	37.3 (0‒87.5)	0.722
	p	*0.437*	*0.032*	
iNKT CD4+/IL6	Proliferative	4.17 (0‒14.9)	0 (0‒1.89)	0.250
	Secretory	2.98 (1.13‒4.83)	1.59 (0‒6.19)	0.667
	p	*0.857*	*0.543*	
iNKT CD4+/IL10	Proliferative	3.01 (0‒7.46)	1.82 (0‒3.77)	0.571
	Secretory	2.32 (1.93‒2.71)	3.31 (0‒20)	0.400
	p	*0.381*	*0.260*	
iNKT CD4+/IL17	Proliferativa	14.8 (8.3‒16)	8.6 (3.8‒16.4)	0.786
	Secretora	11.7 (6.6‒16.9)	9.8 (0‒17.5)	0.711
	p	*>0.999*	*>0.999*	
iNKT CD8+/IL6	Proliferative	4.52 (0‒16.8)	0 (0‒10.7)	0.571
	Secretory	5.74 (1.27‒10.2)	0.8 (0‒10)	0.308
	p	*>0.999*	*0.808*	
iNKT CD8+/IL10	Proliferative	3.85 (1.32‒9.8)	7.14 (0‒9.52)	>0.999
	Secretory	2.68 (2.53‒2.82)	3.01 (0‒11.1)	0.923
	p	*0.857*	*0.639*	
iNKT CD8+/IL17	Proliferative	6.15 (4.07‒17.6)	13.3 (9.52‒14.3)	0.250
	Secretory	10.8 (9.6‒12)	3.95 (0‒54.5)	0.308
	p	*0.381*	*0.139*	
iNKT DN/IL6	Proliferative	1.28 (0‒7.66)	2.27 (0‒3.45)	0.786
	Secretory	0.61 (0.48‒3.57)	0 (0‒1.96)	0.217
	p	*>0.999*	*0.142*	
iNKT DN/IL10	Proliferative	0.52 (0‒1.02)	0 (0‒2.27)	>0.999
	Secretory	0.19 (0‒0.3)	0 (0‒2.44)	0.573
	p	*0.571*	*0.836*	
iNKT DN/IL17	Proliferative	10.2 (5.2‒13.9)	13.6 (3.2‒13.8)	>0.999
	Secretory	14 (8.1‒21.4)	7.1 (0‒13.8)	0.049
	p	*0.393*	*0.446*	

Mann-Whitney test; Data described as median (minimum‒maximum).

### CD25 and CCR7 expression in CD4^+^ iNKT and DN iNKT cells

The number of cells expressing CD25 was similar between the endometriosis and control groups in both the CD4^+^ iNKT (33.5 ± 23.1 vs. 25.8 ± 18.5; *p* = 0.168) and the DN iNKT subsets (39.7 ± 31.8 vs. 33.8 ± 29.8; *p* = 0.416) (Table 2).

**Fig. 4:**
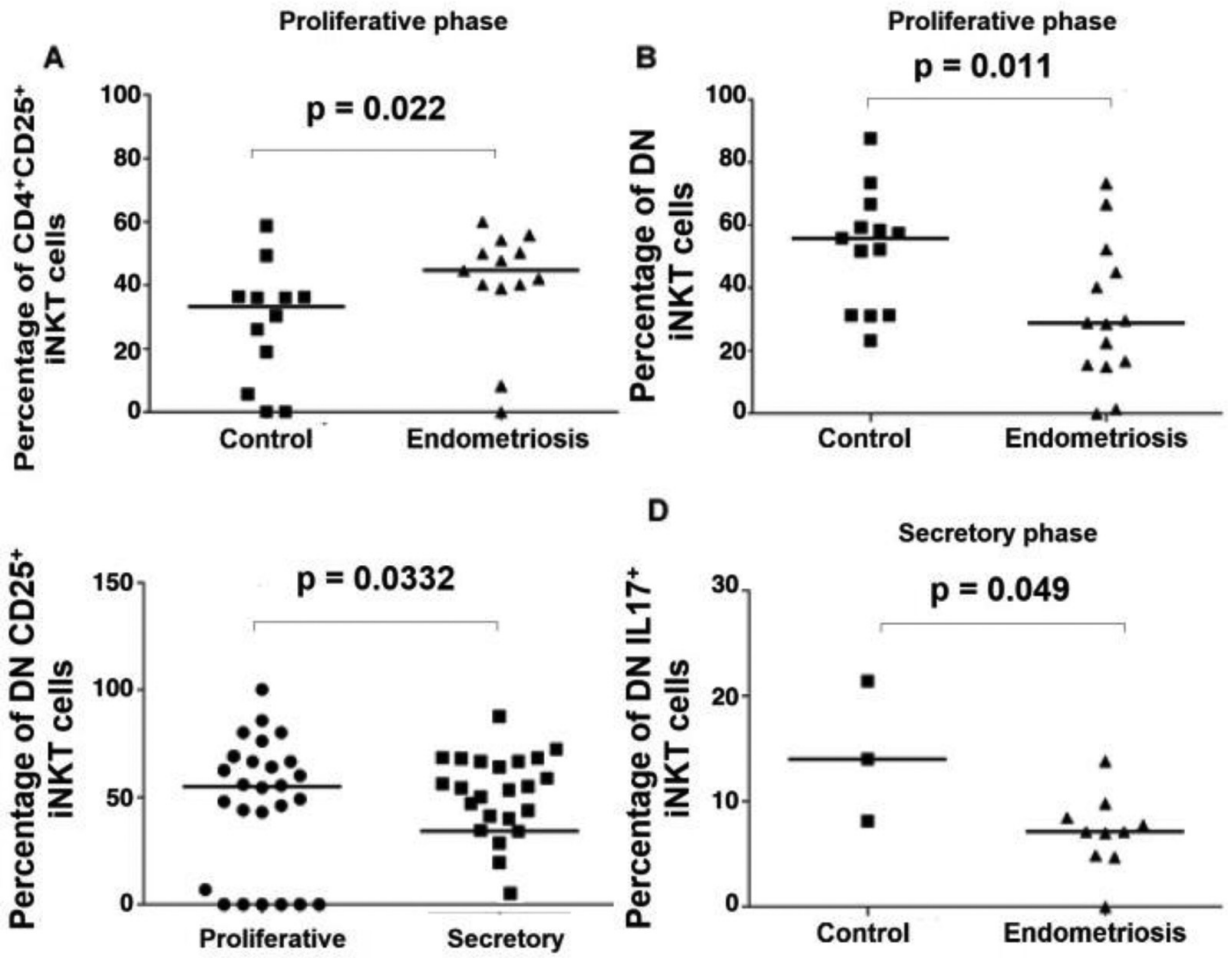
Distribution of frequencies of iNKT cell subsets among menstrual phases. (A) The number of iNKT CD4+ CD25+ cells was increased in the proliferative phase in patients with endometriosis compared to control group; (B) The number of DN CD25+ iNKT cells was increased in the proliferative phase compared to the secretory phase of the menstrual cycle in patients with deep endometriosis; (C) The number of DN iNKT cells was decreased in the proliferative phase in patients with endometriosis compared to the control group; (D) The number of DN IL17+ iNKT cells was decreased in the secretory phase in patients with endometriosis compared to the control group.

When comparing the menstrual phase and iNKT subtypes, the number of CD4^+^ iNKT cells expressing CD25 in the proliferative phase was increased in the endometriosis group compared with the control group (44.6 [0–60] vs. 33.2 [0–58.6]; *p* = 0.022) (Table 3; Fig. 4A). The number DN iNKT cells expressing CD25 was higher in patients with endometriosis in the proliferative menstrual phase than that in the secretory phase (*p* = 0.032) (Table 3, Fig. 4B). There was no significant difference in a total number of iNKT cells and their subsets expressing CD25 in patients with endometriosis and severe pain symptoms compared to those with mild pain symptoms (Tables 4 and 5).

**Table 4:** Number of total iNKT cells, its subsets and cytokine expression in the peripheral blood of patients with mild and severe dysmenorrhea.

	Dysmenorrhea		
Variable	< 7	≥ 7	*p*
	(*n* = 17)	(*n* = 30)	
Total iNKT (mean ± SD)	0.08 ± 0.1	0.23 ± 0.69	0.538
iNKT CD4+	38.4 ± 23.6	36.1 ± 29.8	0.613
iNKT CD4+/CCR7	51 ± 21.7	26.9 ± 31.8	**0.022**
iNKT CD4+/CD25	31.2 ± 21.7	34.9 ± 24.3	0.547
iNKT DN	27.5 ± 23.7	39.3 ± 24.3	0.098
iNKT DN/CCR7	12.67 ± 15.57	10.27 ± 16.28	0.330
iNKT DN/CD25	48.1 ± 28.5	35 ± 33.1	0.193
iNKT CD4+/IL6	1 ± 1.17	1.61 ± 2.4	>0.999
iNKT CD4+/IL10	3.21 ± 2.67	5.02 ± 6.76	0.925
iNKT CD4+/IL17	4.3 ± 4	11.1 ± 4.7	**0.038**
iNKT CD8+/IL6	3.87 ± 5.19	3.2 ± 3.97	0.835
iNKT CD8+/IL10	5.64 ± 4.5	3.91 ± 3.63	0.461
iNKT CD8+/IL17	14.11 ± 22.78	9.26 ± 7.61	0.593
iNKT DN/IL6	0.65 ± 0.92	0.77 ± 1.25	0.867
iNKT DN/IL10	0.2 ± 0.41	0.55 ± 1.03	0.706
iNKT DN/IL17	5.4 ± 4.2	8.9 ± 4	0.246

Mann-Whitney test; SD, Standard Deviation.

**Table 5:** Number of total iNKT cells, its subsets and cytokine expression in the peripheral blood of patients with mild and severe acyclic pelvic pain.

	Acyclic pelvic pain		
Variable	< 7	≥ 7	*p*
	(*n* = 38)	(*n* = 9)	
Total iNKT (mean ± SD)	0.19 ± 0.6	0.08 ± 0.09	0.593
iNKT CD4+	36.8 ± 27.1	38.2 ± 31.7	0.958
iNKT CD4+/CCR7	34.4 ± 30.1	42.2 ± 35.1	0.635
iNKT CD4+/CD25	32.4 ± 23.4	39.5 ± 22.9	0.493
iNKT DN	34.4 ± 25.1	36.2 ± 22.4	0.851
iNKT DN/CCR7	11.81 ± 16.94	8.07 ± 9.01	>0.999
iNKT DN/CD25	39.7 ± 31.8	39.9 ± 34.8	0.894
iNKT CD4+/IL6	0.74 ± 1.02	3.89 ± 3.25	0.267
iNKT CD4+/IL10	4.8 ± 6.5	3.18 ± 1.13	0.921
iNKT CD4+/IL17	7 ± 5.2	13.1 ± 3.9	**0.048**
iNKT CD8+/IL6	3.72 ± 4.83	2.73 ± 2.76	0.945
iNKT CD8+/IL10	5.2 ± 4.24	2.84 ± 2.44	0.454
iNKT CD8+/IL17	13.27 ± 16.36	5.31 ± 2.92	0.539
iNKT DN/IL6	0.93 ± 1.3	0.31 ± 0.35	0.710
iNKT DN/IL10	0.34 ± 0.77	0.67 ± 1.18	0.503
iNKT DN/IL17	8.3 ± 5	6.7 ± 1.3	0.503

Mann-Whitney test; SD, Standard Deviation.

The number of DN iNKT cells was decreased in the proliferative menstrual phase in patients with endometriosis compared to that in the control group (28.8 [0–73.3] vs. 55.8 [23.1‒87.5]; *p* = 0.011) (Table 3, Fig. 4C).

The number of CD4^+^ iNKT cells expressing CCR7 (35.6 ± 30.6 vs. 31.6 ± 31.0; *p* = 0.439) and the number of DN iNKT expressing CCR7 (11.18 ± 15.87 vs. 6.76±14.08; *p* = 0.801) were similar between the endometriosis and control groups (Table 2). There was no significant difference between menstrual cycle phases and a total number of iNKT and their subsets expressing CCR7between the endometriosis and control groups (Table 3). The number of CD4^+^ CCR7^+^ iNKT cells decreased in patients with endometriosis and severe dysmenorrhea compared to patients with mild/absent dysmenorrhea (26.9 ± 31.8 vs. 51.0 ± 21.7; *p* = 0.022) (Table 4). There was no significant difference in the number of iNKT cells and their subsets expressing CCR7 in relation to other pain symptoms.

### Cytokine profile of CD4^+^, CD8^+^ and DN iNKT cells

The number of iNKT DN cells expressing IL-17 was lower in patients with endometriosis compared to the control group (7.8 ± 4.2 vs. 11.5 ± 5.0; *p* = 0.05). There were no differences in the cytokine profiles of the other iNKT subsets (Table 6).

**Table 6:** Number of iNKT subsets and cytokine expression in the peripheral blood of patients with endometriosis and controls.

	Group			
	Control	Endometriosis	Total	
Variable	(*n* = 26)	(*n* = 47)	(*n* = 73)	*p*
iNKT CD4+/ IL-6 (mean ± SD)	5.55 ± 5.71	1.37 ± 1.94	3.09 ± 4.34	0.081
iNKT CD4+/ IL-10	3.08 ± 2.26	4.36 ± 5.51	3.86 ± 4.48	0.856
iNKT CD4+/ IL-17	13 ± 4	8.7 ± 5.5	10.4 ± 5.3	0.160
iNKT CD8+/ IL-6	6.37 ± 5.8	3.44 ± 4.25	4.42 ± 4.89	0.196
iNKT CD8+/ IL-10	3.81 ± 2.91	4.53 ± 3.88	4.29 ± 3.53	0.881
iNKT CD8+/ IL-17	9.11 ± 4.65	10.99 ± 14.18	10.36 ± 11.75	0.455
iNKT DN/ IL-6	1.99 ± 2.57	0.74 ± 1.12	1.21 ± 1.86	0.127
iNKT DN/ IL-10	0.35 ± 0.39	0.44 ± 0.88	0.41 ± 0.72	0.356
iNKT DN/ IL-17	11.5 ± 5	7.8 ± 4.2	9.2 ± 4.8	**0.050**

Mann-Whitney test; SD, Standard Deviation.

Concerning the menstrual cycle phases, the authors observed that the number of DN iNKT cells expressing IL-17 in the secretory phase was decreased in patients with endometriosis compared to the control group (7.1 [0–13.8] vs. 14.0 [8.1–21.4]; *p* = 0.049) (Fig. 4D). There was no difference between the endometriosis and control groups in any menstrual cycle phase on the number of CD4^+^ iNKT cells expressing IL-17 (9.8 [0‒17.5] vs. 11.7 [6.6‒16.9]; *p* = 0.711) and CD8^+^ iNKT cell expressing IL-17 (3.95 [0‒54.5] vs. 10.8 [9.6‒12]; *p* = 0.308). There was no significant difference in the number of any iNKT cells expressing IL-10 or IL-6 between the groups during any menstrual cycle phase (Table 3).

There was an increased number of CD4^+^ iNKT cells expressing IL-17 in the peripheral blood of patients with endometriosis and severe dysmenorrhea compared to those with mild/absent dysmenorrhea (11.1 ± 4.7 vs. 4.3 ± 4.0; *p* = 0.038) (Table 4). There was also an increased number of CD4^+^ iNKT cells expressing IL-17 in the peripheral blood of patients with endometriosis and severe acyclic pelvic pain compared to those with mild pain (13.1 ± 3.9 vs. 7.0 ± 5.2; *p* = 0.048) (Table 5). No significant differences in the numbers of iNKT subsets expressing IL-17 were observed in patients with dyspareunia, cyclic intestinal, or urinary symptoms. Furthermore, there was no relationship between fertility status and the frequency of iNKT cells and subtypes between groups.

### Presence of CD4^+^ iNKT cells in deep endometriosis lesions

**Fig. 5:**
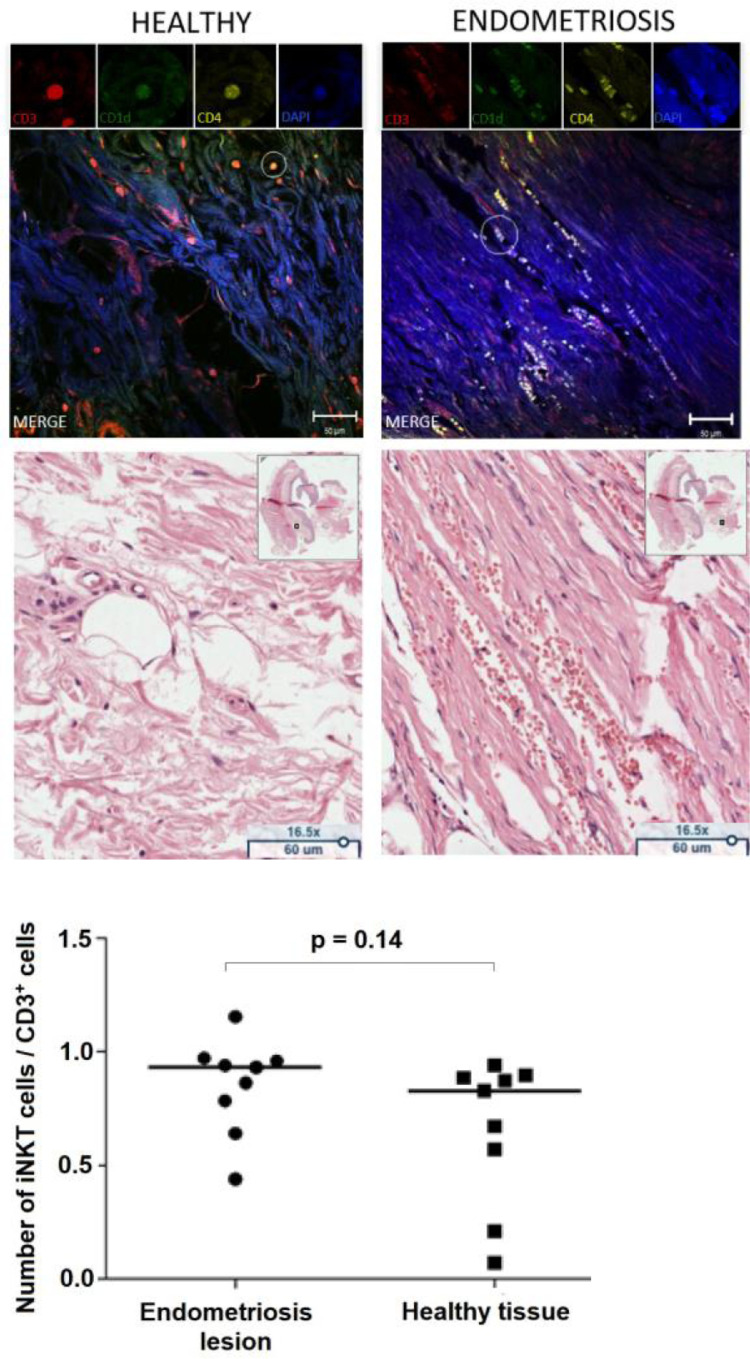
Confocal microscopy of immunostained iNKT cells in endometriosis lesions. (A) Representative overlap of cells stained with antibodies against CD3 (red), CD1d (green), and CD4 (yellow), in healthy regions and areas with endometriosis. Nuclei are counterstained with DAPI (blue). (B) Figure representing the percentage of iNKT cells in the healthy tissues and regions with endometriosis. There was no significant difference between groups (*p* = 0.14).

The authors performed immunofluorescence staining of iNKT cells for the markers CD3, CD4, and CD1d (counterstained with DAPI) in endometriosis lesions and healthy peritoneum from nine patients (Fig. 5A). iNKT cells were present in both endometriosis lesions and healthy areas, and there was no significant difference in the number of iNKT cells between them (Fig. 5B).

## Discussion

The role of the immune system in the pathogenesis of endometriosis has been widely demonstrated in the last few decades. Numerous cytokines have been shown to be abnormally expressed, and variable Th1, Th2, and Th17 responses have been observed in patients with endometriosis.^7^^,^^19^ Recently, NKT cells have been shown to regulate inflammatory responses. Type I NKT cells can be divided into five functional subsets and immune responses. A decreased cytotoxic function of NKT cells in patients with endometriosis may prevent ectopic endometrial cells from being eliminated from the peritoneal cavity, contributing to the development and progression of the disease.^3^^,^^12^

This study evaluated iNKT cell frequency and functionality in patients with endometriosis. To our knowledge, this is the first study that described iNKT cells and their subsets in endometriosis. The baseline patient characteristics were similar between groups, except for BMI, which was higher in the control group. These results were in agreement with previous findings of decreased BMI in patients with endometriosis compared to that in healthy women.^20^ As expected, the frequency of patients with severe dysmenorrhea and cyclic dyschezia was significantly higher in the endometriosis group, as expected. Patients with endometriosis are known to have more pain symptoms than healthy controls.

The present study's findings demonstrated a significantly lower frequency of iNKT cells in the peripheral blood of patients with deep endometriosis than in women without endometriosis. A lower frequency of iNKT cells has been observed in several diseases in which the immune response is dysregulated, including HIV and HTLV infection, common variable immunodeficiency, autoimmune diseases, and some cancers.^13^^,^^15^^,^^21^^,^^22^ Under different pathological conditions, iNKT cells can have either a protective or harmful role, as they have both classically innate and adaptive immunologic characteristics. In endometriosis, a decreased frequency of iNKT cells may impair local immune surveillance and facilitate ectopic implantation in the endometrium.^13^

Estrogen and progesterone control endometrial functions by regulating the expression of thousands of genes during the menstrual cycle.^23^ Different profiles of inflammatory cell frequencies and cytokine secretion have been observed in the peripheral blood, peritoneal fluid, and urine, depending on the menstrual cycle phase.^24^ These data suggest the essential role of sex steroid hormones in the physiology of the immune microenvironment.^25^^,^^26^ The authors showed that the number of iNKT cells was decreased in the secretory phase in patients with deep endometriosis compared to those without endometriosis. This difference could be related to abnormalities in progesterone secretion and sensitivity in patients with endometriosis. Previous studies have shown that progesterone receptor resistance is associated with endometriosis development and persistence.^27^^,^^28^ Abnormalities in progesterone physiology were directly linked to modifications of the immune environment in the topic and ectopic endometrium. Increased levels of estradiol observed in women with endometriosis may also affect NKT cell cytotoxicity and local immune surveillance.^13^ Therefore, the authors hypothesized that the decrease in the number of iNKT cells in patients with endometriosis may be related to an imbalance between estrogen and progesterone levels, which is frequently associated with the disease.

By evaluating iNKT cell subsets, the authors observed a decrease in the number of DN iNKT cells, the most predominant iNKT subtype in humans, which have effector functions in immune responses.^18^^,^^29^ In 2002, Lee et al.^18^ showed that CD4^+^ iNKT cells exclusively expressed the interleukin-2 alpha chain (CD25) in healthy individuals. The authors observed that more DN iNKT cells expressed CD25, compared with CD4^+^ iNKT cells. These results suggest that the expression of CD25 in DN iNKT cells is downregulated in patients with endometriosis. Previous studies demonstrated that in healthy individuals, TH1-like type I NKT cells, most of them DN NKT cells, produce TH1-associated cytokines such as IFN-γ and TNF-α upon stimulation, and therefore, may exert limited cytotoxic function.^30^

The authors also compared iNKT cell frequencies in the peripheral blood of patients with endometriosis according to pain intensity and fertility status. The authors observed a decreased number of CD4^+^ CCR7^+^ iNKT cells in patients with endometriosis and severe dysmenorrhea, suggesting that the immune response may play a role in the severity of the disease and its symptoms. In 2012, Guo et al.^31^ also observed lower NKT cell percentages and IFN-γ and IL-4 levels in the peripheral blood and peritoneal effusions of 60 patients with endometriosis compared with 20 healthy controls. They showed that the number of NKT cells, as well as IFN-γ, and IL-4 levels, were inversely correlated with endometriosis stage, supporting the correlation between the number of NKT cells and the severity of endometriosis.

Evidence suggests that iNKT cell subpopulations (CD4^+^, CD8^+^, and CD4^−^CD8^−^) produced different profiles of cytokine secretion and activation of NK and B cells, leading to different Th responses.^32^ In 2011, O'Reilly et al.^33^ observed a differential secretion pattern of cytokines after stimulation of CD4^+^, CD8^+^, and CD4^−^CD8^−^ iNKT cells. Several studies have demonstrated variations in the frequency and function of iNKT cell subsets in patients with different diseases.^13^^,^^34^ DN iNKT cells have an essential Th1 response pattern, releasing higher amounts of IFN-γ and TNF-α after stimulation.^35^ Accordingly, the present study's findings demonstrate a decreased percentage of DN iNKT in patients with endometriosis. Since DN iNKT cells produce a Th1 response and a balance in Th1/Th2 responses is essential for immune homeostasis, the authors hypothesized that this abnormality is implicated in the pathophysiology of endometriosis.^7^^,^^19^

The authors demonstrated that iNKT DN IL-17^+^ cells are in present lower proportions in patients with endometriosis than in women without endometriosis. In contrast, the authors also showed an increased frequency of CD4^+^ IL-17^+^ iNKT cells in patients with endometriosis with severe dysmenorrhea and with severe acyclic pelvic pain. IL-17 is a member of a family of cytokines predominantly produced by activated CD4^+^ T-cells. It has potent pro-inflammatory properties and is involved in the modulation of the immune response in inflammatory disorders and pain.^36^ Furthermore, IL-17 seems to be implicated in the development of endometriosis by inducing estrogen production, endometriotic stromal cell proliferation, and secretion of inflammatory mediators.^4^^,^^37^ Previous studies have shown that increased levels of IL-17 are involved in visceral and neuropathic pain.^36^^,^^38^ The abnormal frequencies of CD4^+^ IL-17^+^ iNKT cells observed in the present study may be, in part, responsible for endometriosis-related pain symptoms.

The results of the present study led us to hypothesize that iNKT cells and their subtypes may play an essential role in the pathogenesis of endometriosis. Abnormalities in the frequency of iNKT cells may impair the proper functioning of the immune system, allowing the implantation and proliferation of endometriosis lesions.

Currently, most treatments available for endometriosis are hormonal medications, which also work as contraceptives. iNKT cells may be a target for the development of new non-hormonal drugs that may be important for women who are trying to conceive or have any contraindication to the use of hormones.^3^ Since other studies have recently demonstrated a positive effect of immunotherapy by activating iNKT cells with different antigens in liver disease, autoimmune diseases, and antitumor therapy, it is possible to use this treatment strategy for other inflammation-related disorders such as endometriosis.^39^

## Conclusion

In conclusion, the frequency of total iNKT and DN iNKT cells was decreased in patients with endometriosis. Patients with endometriosis with severe dysmenorrhea and acyclic pelvic pain had increased production of IL-17 by CD4^+^ iNKT cells and decreased numbers of CD4^+^ CCR7^+^ cells. Further studies in animal models could use targeted drugs to enhance or inhibit the activity of iNKT cells and further confirm these results, aiming to develop new therapeutics for endometriosis. Overall, these results suggest that iNKT cells play a role in the pathogenesis of endometriosis and can be exploited in the development of new diagnostic and therapeutic agents.

## Authors’ contributions

Correa FJS study design, collection of data, analysis of results, manuscript drafting. Andres MP and Abrão MS study design, analysis of results, manuscript drafting. Rocha TP analysis of results, manuscript drafting. Carvalho AEZ and Carvalho KI study design, collection of data. TPA Aloia collection of data. Corpa MVN collection of data, analysis of results. Kallas EG and Baracat EC study design, manuscript drafting. Mangueira LP collection of data, analysis of results.

## Funding

This study was supported by Fundação de Amparo à Pesquisa do Estado de São Paulo (FAPESP # 2012/05425-7).

## Ethics approval

This research was approved by the institutional ethics committee of Hospital das Clinicas, Faculdade de Medicina, Universidade de São Paulo (CAPPesq 235869/13) and performed in accordance with the ethical standards of the 1964 Declaration of Helsinki.

## Availability of data and material

All the data and materials used in this research are available upon request.

## Code availability

Not applicable.

## Consent to participate

Informed consent was obtained from all study participants.

## Consent for publication

Not applicable.

## Conflicts of interest

The authors declare no conflicts of interest.
